# Attractor Stability of Boolean networks under noise

**Published:** 2025-06-18

**Authors:** Byungjoon Min, Jeehye Choi, Reinhard Laubenbacher

**Affiliations:** 1 Department of Physics, Chungbuk National University, Cheongju, Chungbuk 28644, Korea; 2 Advanced-Basic-Convergence Research Institute, Chungbuk National University, Cheongju, Chungbuk 28644, Korea; 3 Department of Medicine, University of Florida, Gainesville, FL 32610, USA

## Abstract

We study the impact of noise on attractor dynamics in Boolean networks, focusing on their stability and transition behaviors. By constructing attractor matrices based on single-node perturbations, we propose a framework to quantify attractor stability and identify dominant attractors. We find that attractors are more stable than predicted by basin sizes, showing the importance of dynamical structure in noisy environments. In addition, under global perturbations, basin sizes dictate long-term behavior; under local noise, however, attractor dominance is determined by noise-induced transition patterns rather than basin sizes. Our results show that transition dynamics induced by stochastic perturbations provide an efficient and quantitative description for the attractor stability and dynamics in Boolean networks under noise.

## I. INTRODUCTION

Boolean networks have been widely used as mathematical models for various biological systems, in particular the study of gene regulatory networks [1–3], metabolic networks [4], signal transduction networks [5], and neural networks [6]. These models provide a simple yet powerful framework to capture the underlying interactions and dependencies between components [7]. In the classical Boolean networks, each node represents a binary state (0 or 1), and the state of each node is updated based on a Boolean function that depends on the states of its neighboring nodes [1, 8, 9]. Many variants of Boolean network models have since been proposed to better reflect biological complexity, and a wide range of dynamical behaviors have been extensively studied [10–19]. One of the key features of Boolean networks is that their state trajectories eventually converge to stable configurations (fixed points) or recurring patterns (limit cycles), so called “attractors” [1, 20, 21]. Attractors play a crucial role in understanding the long-term behavior of the system, as they correspond to different functional states in biological systems [1, 11].

Noise in biological systems is ubiquitous, arising from intrinsic fluctuations in biochemical reactions and external environmental variations [22–24]. Noise can randomly alter the states of biological systems, independent to the system’s underlying rules and thus plays a significant role in shaping dynamics [22, 24–26]. In Boolean networks, noise can be implemented as random state flips of individual nodes, that are independent of the Boolean update rules [25–28]. From the perspective of attractor dynamics, such noise can induce significant changes: rather than remaining permanently trapped in a single attractor, the system may transition between different attractors over time [25, 27, 29]. As a result, understanding how attractors behave under noise is essential for capturing the full dynamical properties of biological systems [29]. It also allows for a more complete view of biological stability and variability under noisy environments. A related modeling framework is the probabilistic Boolean networks [3, 30], where state transitions are governed by probabilities rather than deterministic rules, sharing similar notions of stochastic effects in Boolean networks. In contrast to the probabilistic framework, where randomness is incorporated into the update rules, the noise considered here operates independently of the system’s deterministic rules.

Given the importance of understanding how noise influences attractor dynamics, it is crucial to analyze the stability of attractors under noise and the long-term dynamics of Boolean networks. Among existing approaches, Derrida analysis has been widely used to capture the spread of perturbations by assessing the average sensitivity in Boolean networks [20, 31–33]. However, this method is limited in its ability to address noise-driven transitions between attractors, as it does not explicitly include stochastic effects and, moreover, operates at the node level rather than the attractor level [28]. To this end, we propose a framework for computing transition probabilities between attractors under noise. This framework enables us to systematically assess the stability of attractors in Boolean networks and understand how noise impacts their long-term dynamics.

The remainder of this paper is organized as follows. In Sec. II, we begin by introducing Boolean networks under noise. Next, in Sec. III, we describe the mathematical framework for quantifying transition probabilities between attractors and present our approach for constructing transition matrices to analyze the transition patterns of attractors. In Sec. IV, we analyze how noise alters the long-term behavior of Boolean networks by quantifying attractor stability, identifying dominant attractors, and characterizing the structure of noise-induced transitions. We also apply our analysis to real-world Boolean networks to examine the relevance of our findings in biological systems. Finally, in Sec. V, we summarize our findings and discuss the implications of our results and potential directions for future research.

## II. BOOLEAN NETWORKS UNDER NOISE

We consider a Boolean network which consists of N nodes. Each node i has a binary state $x_{i}\in \left\{0,1\right\}$, and the state of the system at time t is given by the vector $\text{x}\left(t\right)=\left(x_{1}\left(t\right),x_{2}\left(t\right),\cdots ,x_{N}\left(t\right)\right)$. The evolution of the system follows a deterministic update rule:

(1) $$\text{x}\left(t+1\right)=F\left(\text{x}\left(t\right),t\right),$$

where F is a Boolean function that governs the dynamics of nodes based on the states of its input nodes at time t. The Boolean function F is predefined and remains fixed throughout the evolution of the system. The system updates all nodes in parallel at each time step. Since the dynamics are deterministic, the system eventually converges to a single attractor in any finite system. These attractors can be either fixed points where the state remains a single state or limit cycles where the system oscillates among a finite set of states [8].

**FIG. 1.**
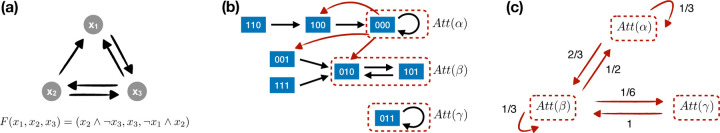
(a) Wiring diagram of a Boolean network and its Boolean function are shown. (b) State space of the Boolean network and its attractors are identified. A random flip of a node due to local noise results in the transition of the state (0, 0, 0), as illustrated in the diagram. (c) The probabilities of remaining within the same attractor or transitioning to a different attractor due to local noise are illustrated.

An example of a Boolean network with N=3 is depicted in Fig. 1(a). The wiring structure shows regulatory influences between nodes through directed edges, with Boolean functions specifying how each node updates its state based on its inputs. The corresponding state space of the network is shown in Fig. 1(b), where each node represents a possible configuration of the system and directed edges indicate deterministic evolution between states according to the Boolean update rules. As shown in Fig. 1(b), all trajectories eventually lead to one of the attractors, which can be either a fixed point or a limit cycle.

We introduce stochastic effects by adding noise to the system in the form of random flipping of the state of a node. Under noisy conditions, the system evolves through a combination of deterministic Boolean rules and stochastic perturbations. When noise occurs, the state of an affected node is randomly altered, leading to deviations from the purely deterministic trajectory [25, 26, 34, 35]. Such fluctuations can drive the system to transition between attractors, preventing it from remaining in a single attractor indefinitely. In this study, we assume that noise occurs relatively infrequently. If noise were too frequent, the system would no longer follow the logic of a Boolean network but instead behave like a randomly fluctuating system, which would lose biological relevance.

Our main interest in this model lies in how noise affects attractor dynamics in Boolean networks. To be specific, we examine the stability of individual attractors by analyzing how likely the system is to remain within the same attractor after random perturbations. We also explore which attractors tend to dominate the steady-state behavior of the system under noise, by characterizing the transition patterns between attractors and quantifying the probabilities of noise-induced transitions.

## III. TRANSITION MATRICES OF BOOLEAN NETWORKS UNDER NOISE

In this section, we propose a framework for constructing a transition matrix that encodes the probabilities of transitions between attractors due to noise. Using this matrix, we analyze the stability of attractors and the consequences of transitions between them in noisy environments.

### A. Transition probabilities between attractors

The process for computing the transition matrix under noise is as follows. We first simulate the time evolution of states in a given Boolean network and identify all possible attractors. Each state in the network converges to one of these attractors after passing through transient states. We introduce noise by flipping the value of a single randomly chosen node in a given attractor state, and simulate the dynamics to determine the resulting attractor. This process mimics external perturbations that disrupt the deterministic evolution of the system. Since noise acts intermittently, the Boolean network remains in attractors for most of the time rather than in transient states. Therefore, we restrict our analysis to attractor states, as the system stays in them with high probability. By repeating this process for every state in each attractor, we estimate the transition probabilities between attractors.

The specific transition probability from attractor α to attractor β is computed as follows. Let $\ell_{\alpha }$ be the length of attractor α, meaning that it consists of $\ell_{\alpha }$ states. For each state in α, we flip each of the N nodes once, one flip per node, and count the number of instances $\nu_{\alpha \beta }$ in which the system transitions to attractor β. This results in a total of $N\ell_{\alpha }$ flipping attempts across all states in attractor α. When $\nu_{\alpha \beta }$ transitions occur from attractor α to attractor β out of $N\ell_{\alpha }$ trials, the corresponding transition probability is defined as

(2) $$m_{\alpha \beta }=\frac{\nu_{\alpha \beta }}{N\ell_{\alpha }}.$$

Note that the system may remain in the same attractor after a flip, which corresponds to $m_{\alpha \alpha }$.

A schematic illustration of a single-node flip is shown in Fig. 1(b). For example, consider the attractor state (0, 0, 0). In this case, flipping one of the three nodes results in the states (1, 0, 0), (0, 1, 0), and (0, 0, 1), each of which occurs with equal probability 1/3 under noise. By repeating this procedure for all states in every attractor, we estimate the transition probabilities between attractors. The resulting attractor-to-attractor transitions are represented as a directed network, shown in Fig. 1(c), where nodes correspond to attractors and edge weights indicate the transition probabilities induced by noise.

### B. Stability and dominance of attractors from transition matrices

From the computed transition probabilities, we construct a transition matrix

(3) $$M=\left(\begin{array}{cccc} m_{11} & m_{12} & \cdots & m_{1n}\\ m_{21} & m_{22} & \cdots & m_{2n}\\ \vdots & \vdots & \ddots & \vdots \\ m_{n1} & m_{n2} & \cdots & m_{nn} \end{array}\right)$$

where each element $m_{\alpha \beta }$ represents the probability of transitioning from attractor α to attractor β under noise. The matrix M defines a Markov chain over the set of attractors, where transitions are governed by noise-induced perturbations. Each element $m_{\alpha \beta }$ represents the probability of transitioning from attractor α to attractor β, satisfying $0\leq m_{\alpha \beta }\leq 1$ and $\sum_{\beta }m_{\alpha \beta }=1$ for all α.

The diagonal elements $m_{\alpha \alpha }$ represent the probability that the system remains in the same attractor α after flipping a node. We interpret this quantity as a measure of attractor stability; that is, higher values indicate more robust attractors, whereas lower values imply a higher tendency to transition to other attractors under noise. The off-diagonal elements $m_{\alpha \beta }$ with α≠β quantify the probabilities of noise-induced transitions between different attractors. Thus, the transition matrix M captures the overall stability landscape of the attractor structure in the presence of noise.

In addition to stability, M also provides insight into the dominance of attractors through its long-term stationary distribution. The transition matrix has a principal eigenvalue $\lambda_{1}=1$ with a corresponding eigenvector $\vec{v}$ satisfying $M\vec{v}=\vec{v}$. The components of $\vec{v}$ form the stationary distribution over attractors, where $v_{\alpha }$ represents the fraction of time the system spends in attractor α. In this sense, attractors with larger $v_{\alpha }$ are considered dominant, as the system tends to stay in them more frequently under noise.

### C. Global randomization and attractor basins

To set a baseline for comparison, we consider a global perturbation scenario in which noise simultaneously and randomly alters the states of all nodes. In this case, the entire state rather than a single node undergoes a random change. Specifically, each node in the Boolean network is independently assigned 0 or 1 with equal probability when noise is applied. We refer to this scenario as “global randomization”, as flipping is applied to the entire network rather than to individual nodes. It is important to note that this type of perturbation is fundamentally different from the local noise model considered in our study, where only a single node’s state is flipped. The global randomization erases all memory of the previous state since the system is entirely reset to a random point in the state space.

In this setting, the transition matrix is determined solely by the basin sizes of the attractors. Here, the “basin” of an attractor refers to the set of all initial states that converge to that attractor, and its relative size, b, reflects how likely a randomly selected initial state converges to that attractor. Since global randomization selects states uniformly at random from the full state space, the probability $m_{\alpha \beta }$ that global noise drives the system from attractor α to attractor β depends solely on the basin size $b_{\beta }$ of β. To be specific, $m_{\alpha \beta }=b_{\beta }/\left(N\ell_{\alpha }\right)$, where $b_{\beta }$ denotes the normalized basin size of attractor β. As a result, all columns of the matrix M are identical, and the eigenvector associated with the principal eigenvalue is given by the basin size distribution, $b_{\alpha }$.

Therefore, under global noise, attractors with larger basins exhibit higher probabilities of being reached, and basin size becomes the primary indicator of long-term behavior in this scenario. This observation provides a mathematical rationale for the use of basin size as an indicator of attractor importance or stability, as larger basins are generally associated with greater robustness to perturbations [21, 36]. However, this correspondence does not hold under the local noise model considered in our study, where transitions depend on how individual attractor states respond to single-node perturbations. Nevertheless, basin size serves as a baseline for evaluating how local noise modifies attractor dynamics.

## IV. RESULTS

In this section, we analyze the stability of attractors and the probabilities of transitions between them. We then study how noise influences attractor dynamics in random Boolean networks to understand fundamental properties. To this end, we generate random regular networks with degree k as the underlying structures, and assign Boolean functions randomly according to a given bias parameter p, which represents the probability that the output of a Boolean function is 0. For comparison across different values of k, we use the average sensitivity $s=2p\left(1-p\right)k$ [20, 31] as a unified control parameter. The sensitivity s quantifies the expected number of node state changes caused by flipping a single input and serves as a standard measure of the system’s dynamical sensitivity. We also apply our analysis to empirical networks to explore the real-world implications.

### A. Stability of attractors under noise

In this section, we study the stability of attractors in Boolean networks by analyzing the system’s ability to return to the same attractor after a perturbation caused by local noise. In this sense, the diagonal elements $m_{\alpha \alpha }$ of the transition matrix represent the stability of attractor α. To assess attractor stability at the network level, we compute the trace of the transition matrix, $\text{Tr}\left(M\right)$. A larger trace indicates that attractors in Boolean networks are more stable to noise. As a baseline for comparison, we consider global randomization in which the system’s state is reset entirely at random. In this null model, the trace of the transition matrix equals one by definition, as it results from the normalization of basin sizes. We therefore use $\text{Tr}\left(M\right)=1$ as a reference point, and evaluate the stability of Boolean networks by measuring their trace values.

**FIG. 2.**
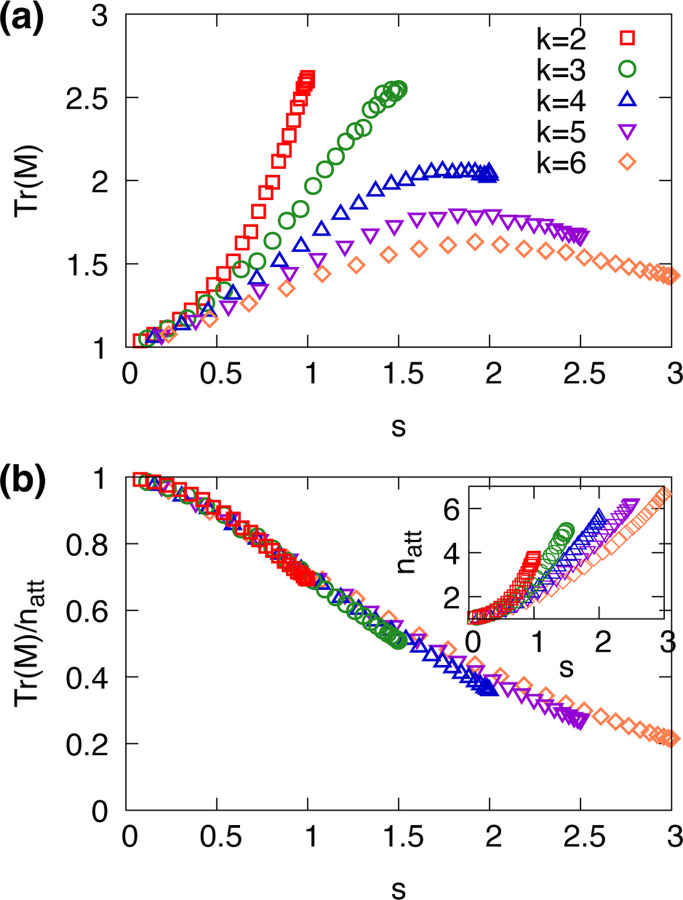
(a) The trace $\text{Tr}\left(M\right)$ of transition matrix for random Boolean networks on random regular networks with size N=20 and various degrees k with respect to the sensitivity s is shown. (b) The trace normalized by the number of attractors, $\text{Tr}\left(M\right)/n_{\text{att}}$ is shown. Inset shows the average number $n_{\text{att}}$ of attractors for given k and s.

Our results in Fig. 2(a) show that the trace $\text{Tr}\left(M\right)$ is consistently greater than unity across various values of the connectivity k and sensitivity s, on random regular networks with N=20. These observations suggest that Boolean networks have an intrinsic resilience to local perturbations that is higher than expected from basin size. While basin size captures the probability of reaching an attractor under global randomization, it is insufficient to account for how the system tends to remain within the same attractor under local noise. Our findings suggest the importance of assessing stability through dynamical measures, $\text{Tr}\left(M\right)$, which more directly reflect the system’s resilience to noise.

In Fig. 2(b), we show the average attractor stability, defined as $\text{Tr}\left(M\right)/n_{\text{att}}$, where $n_{\text{att}}$ is the number of attractors in a given network. This normalization is introduced because the number of attractors varies across different network instances and parameter settings. The results show that average attractor stability tends to decrease as the average sensitivity s increases. Networks with different values of connectivity k but the same value of sensitivity s exhibit similar levels of attractor stability. This indicates that the sensitivity parameter s plays a role as a more reliable predictor of network-level stability than k or p alone.

To understand the source of high attractor stability, we analyze the internal similarity of states within attractors. Consider attractor α, which consists of $\ell_{\alpha }$ binary states $\left\{x_{1}^{\left(\alpha \right)},x_{2}^{\left(\alpha \right)},\ldots ,x_{l_{\alpha }}^{\left(\alpha \right)}\right\}$, where $\ell_{\alpha }$ is the length of the attractor α, i.e., the number of distinct states in the attractor. Each state $x_{i}^{\left(\alpha \right)}$ is a binary vector of length N. The similarity $\sigma^{\alpha }$ for attractor α is defined as the average probability that two randomly selected states in attractor α have the same value at a given position. Let $n_{1}^{\left(\alpha ,i\right)}$ and $n_{0}^{\left(\alpha ,i\right)}$ respectively be the number of ones and zeros in attractor α in a position i. Then the similarity σ can be computed as

(4) $$\sigma_{\alpha }=\frac{1}{N}\sum_{i=1}^{N}\frac{\left(n_{0_{2}}^{\left(\alpha ,i\right)}\right)+\left(n_{1_{2}}^{\left(\alpha ,i\right)}\right)}{\left(\begin{array}{c} l_{\alpha }\\ 2 \end{array}\right)}$$

For fixed-point attractors with length $\ell_{\alpha }=1$, we define the similarity $\sigma_{\alpha }=1$, since all positions are trivially identical. This allows a unified similarity measure for all attractors regardless of their lengths.

To compute the average similarity σ across all attractors in the network, we introduce the following approximation, instead of explicitly computing all binomial coefficients over attractor state. Let $\mu_{i,\alpha }=n_{1}^{\left(\alpha ,i\right)}/\ell_{\alpha }$ be the fraction of states with value 1 at position i. When the attractor length $\ell_{\alpha }$ is large, we approximate the overall similarity of attractors as

(5) $$\sigma \approx \frac{1}{n_{\text{att}}}\sum_{\alpha =1}^{n_{\text{att}}}\left[\frac{1}{N}\sum_{i=1}^{N}\left(\mu_{i,\alpha }^{2}+{\left(1-\mu_{i,\alpha }\right)}^{2}\right)\right].$$

In this approximation, the similarity σ represents the probability that two randomly selected elements are equal, either both 0 with probability ${\left(1-\mu \right)}^{2}$ or both 1 with probability $\mu^{2}$.

**FIG. 3.**
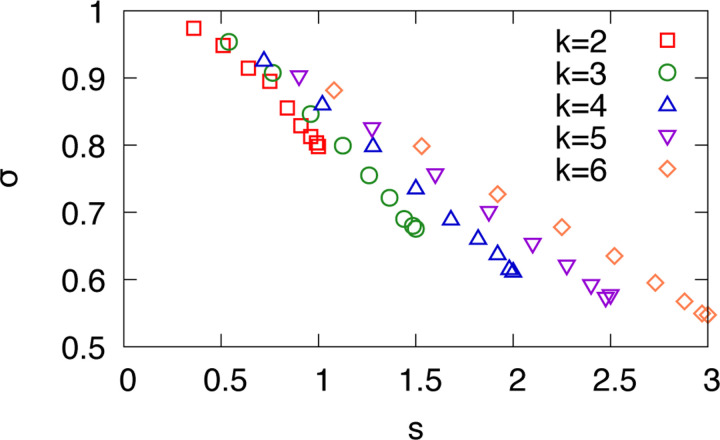
Similarities σ of states within attractors are shown as a function of the sensitivity s. Results are obtained from random regular networks with N=15, and various values of k, averaged over 10^6^ independent runs.

Figure 3 shows the similarity σ of states in an attractor as a function of average sensitivity s, for various values of connectivity k. If attractors were composed of randomly distributed states, σ becomes 1/2. However, the observed values are consistently higher than 1/2, indicating that the states within each attractor tend to be more similar to one another than randomly chosen states. Therefore, local perturbations, i.e., flipping a single node are less likely to push the system out of its attractor and it partially explains why Boolean networks exhibit higher attractor stability under noise. In addition, the pattern of σ in Fig.3 shows a decreasing trend with increasing s. This pattern shares a similarity with that of the average attractor stability $\text{Tr}\left(M\right)/n_{\text{att}}$ shown in Fig.2(b). It suggests that σ provides a useful qualitative indicator of attractor stability in Boolean networks.

### B. Dominance and stationary distribution of attractors

The transition matrix provides a framework for identifying the dynamical importance of attractors under noise. As discussed in Sec. IIB, its principal eigenvector $\vec{v}$ offers a theoretical estimate of the fraction of time spent in each attractor. To validate this prediction, we measure the average duration that the system spends in different attractors of Boolean networks under noise. For each simulation, we track the transitions between attractors and record the time $t_{\alpha }$ spent in each attractor α. Specifically, we measure the time fractions $t_{\alpha }/t_{\text{tot}}$, where $t_{\text{tot}}$ is the total simulation time.

**FIG. 4.**
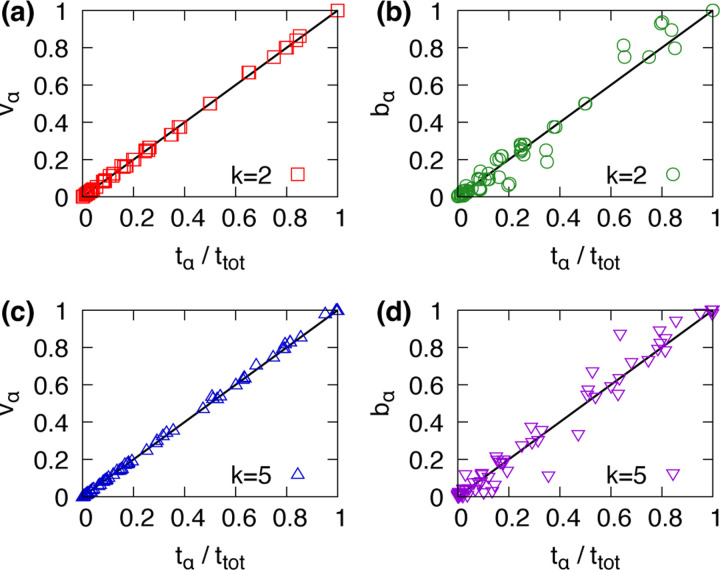
Comparison between the empirically measured time fractions spent in attractors, $t_{\alpha }/t_{\text{tot}}$, and the principal eigenvector components $v_{\alpha }$ of the transition matrix is shown for random regular networks with N=20 and k=2,5. Panels (a,c) show comparisons with eigenvector predictions, while panels (b,d) show comparisons with static basin sizes.

In Fig. 4, we compare the components $v_{\alpha }$ of principal eigenvector with the numerically measured time fractions. Figures 4(a,c) show that the eigenvector accurately predicts the stationary distribution, while Figs. 4(b,d) demonstrate that basin size provides a less accurate estimate of the empirical distribution. The strong agreement between $v_{\alpha }$ and the measured values $t_{\alpha }/t_{\text{tot}}$ confirms that the system’s stationary distribution is well predicted by the leading eigenvector of the transition matrix. These results suggest that the steady state of Boolean networks are governed by noise-induced transition probabilities rather than static basin structures.

Additionally, this correspondence implies that attractor dynamics can be interpreted as a random walk on the attractor transition network, with transitions governed by the probabilities $m_{\alpha \beta }$. This is conceptually related to the PageRank algorithm [37] or eigenvector centrality [38–41], in which the stationary distribution of a Markov chain determines node importance. In our framework, attractors play the role of nodes, and their importance is reflected in the time that the system spends in each under noise predicted theoretically by the leading eigenvector. Thus, this approach provides an efficient way to identify dominant attractors and to predict the asymptotic behavior of Boolean networks under stochastic perturbations.

### C. Entropy of attractor distributions

**FIG. 5.**
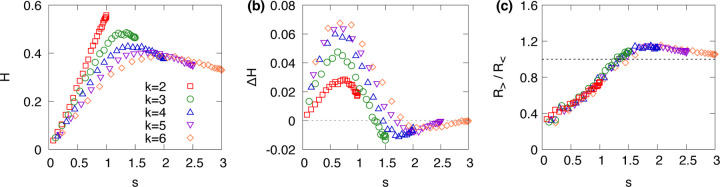
(a) Entropy H of the principal eigenvector as a function of network sensitivity s, (b) the difference ΔH between entropy from basin sizes and from the eigenvector distribution, and (c) the ratio $R_{>}/R_{<}$ comparing transition bias toward larger versus smaller basins. All results are obtained on random regular networks with N=20 and various values of k.

We further study how broadly the system explores the attractor space under noise. To this end, we compute the normalized Shannon entropy H of the principal eigenvector $\vec{v}$ of the transition matrix, defined as

(6) $$H=-\frac{1}{H_{\max}}\sum_{\alpha }v_{\alpha }\log_{2}v_{\alpha },$$

where $v_{\alpha }$ represents the steady-state probability that the system occupies attractor α under noise, and $H_{\max}=\log_{2}n_{\text{att}}$ is the maximum entropy possible for a given number of attractors. The normalization ensures that $H\in \left[0,1\right]$. High entropy indicates that the system frequently visits many attractors with similar probabilities, reflecting broad exploration and dynamical diversity. In contrast, low entropy implies strong localization around a few dominant attractors. Figure 5(a) shows that the Shannon entropy H as a function of s for various k. This result shows a shift from localized to distributed dynamics as s varies.

To assess how this behavior compares with the static structure of the state space, we compute the entropy difference ΔH between the stationary distribution and the basin size distribution:

(7) $$\Delta H=-\frac{1}{H_{\max}}\sum_{\alpha }\left(v_{\alpha }\log_{2}v_{\alpha }-b_{\alpha }\log_{2}b_{\alpha }\right),$$

where $b_{\alpha }$ is the normalized basin size of attractor α. Positive values of ΔH indicate that the system explores a narrower subset of attractors under local stochastic dynamics than would be expected based on basin sizes alone. Figure 5(b) shows that ΔH is generally positive, particularly at low sensitivity, showing that noise-driven dynamics remain localized despite the existence of other attractors. As s increases, ΔH gradually decreases and hovers near zero, suggesting convergence toward the global randomization predicted by basin sizes.

To better understand this localization, we examine transition asymmetries between attractors. Figure 5(c) shows the ratio of transition probabilities between attractors of different basin sizes. We compute the ratio $\frac{m_{\alpha \beta }}{b_{\alpha }}$, where $m_{\alpha \beta }$ is the probability of transitioning from attractor α to β, and $b_{\alpha }$ is the basin size of the source attractor. We then compare this value for transitions into larger basins $\left(R_{>}\right)$ versus smaller ones $\left(R_{<}\right)$, using the ratio $R_{>}/R_{<}$ to quantify the directionality of transitions. Our results indicate that for s<1.5, transitions tend to favor smaller basins, i.e., $R_{>}/R_{<}<1$, suggesting a bias in the system’s exploration. That is, attractors with larger basin sizes become more dominant in this regime, leading to localization around a limited set of attractors. At higher sensitivities, this bias diminishes and the ratio approaches 1, implying that the transition dynamics become more random and basin-size neutral, consistent with global randomization.

### D. Application to real-world Boolean networks

**FIG. 6.**
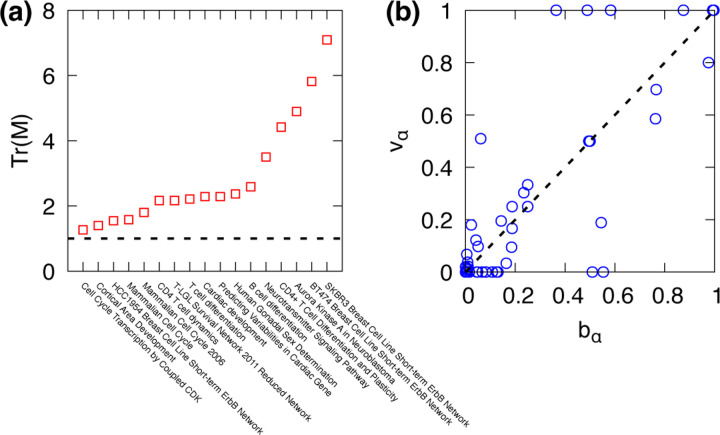
(a) The trace of the transition matrix, $\text{Tr}\left(M\right)$, is shown in descending order for real-world Boolean networks. (b) A comparison between the principal eigenvector components $v_{\alpha }$ and basin sizes $b_{\alpha }$ for all attractors across the real-world networks is presented.

Finally, we apply our framework to real-world Boolean networks obtained from the Cell Collective database [42, 43]. These datasets include biological systems such as gene regulatory networks, signaling pathways, and developmental processes. We found that the trace of the transition matrix, $\text{Tr}\left(M\right)$, exceeded unity consistently across all tested networks as shown in Fig. 6(a). This indicates that, similar to random Boolean networks, real-world Boolean networks exhibit enhanced attractor stability under local stochastic perturbations. This result suggests that high stability to noise is not an artifact of synthetic models but a robust feature observed in biological Boolean systems.

We further compared the stationary distributions predicted by the principal eigenvectors with the basin size distributions. In Fig. 6(b), each data point corresponds to an individual attractor from 17 different real-world Boolean networks, where the horizontal axis represents the normalized basin size and the vertical axis indicates the corresponding component of the principal eigenvector. We found that basin sizes deviate from eigenvector components, indicating that static basin structures are insufficient to predict the long-term dynamics under noise. Since noise is inherent in real-world systems, our framework based on transition dynamics provides a reliable prediction on the behavior of real-world Boolean networks.

## V. DISCUSSION

In this study, we developed a framework to quantify the stability and transition dynamics of attractors in Boolean networks under noise. By constructing and analyzing transition matrices, we showed that attractors are more stable against local noise than predicted by static basin sizes. We also found that basin sizes alone are insufficient to predict long-term dynamics under noise, and that transition dynamics allows us a more reliable predictions. We further demonstrated that real-world biological networks exhibit similar resilience to noise, suggesting that this can be a general feature of biological systems.

Our study provides an alternative perspective on attractors of Boolean networks, showing the importance of dynamical structure and stochastic effects in noisy environments. These findings suggest that noise can induce structured yet nontrivial transitions between attractors, and that Boolean networks exhibit an intrinsic resilience. It remains important to assess the generality of these findings, especially in large-scale networks, as a key direction for future work. In addition, future research could explore how specific topological features, such as modularity [18, 44], redundancy [17, 45], and/or the presence of canalizing functions [14, 15, 46], contribute to this stability. Extending the framework to include asynchronous updates [13], multi-node perturbations [35], or correlated noise [47] may provide further insight into biological function.
